# Supplementary figures and images for: Protective role of protease-activated receptor-2 in anaphylaxis model mice
Source: PLoS One. 2024 Apr 18;19(4):e0283915. doi: 10.1371/journal.pone.0283915 (PMC11025949; doi:10.1371/journal.pone.0283915)

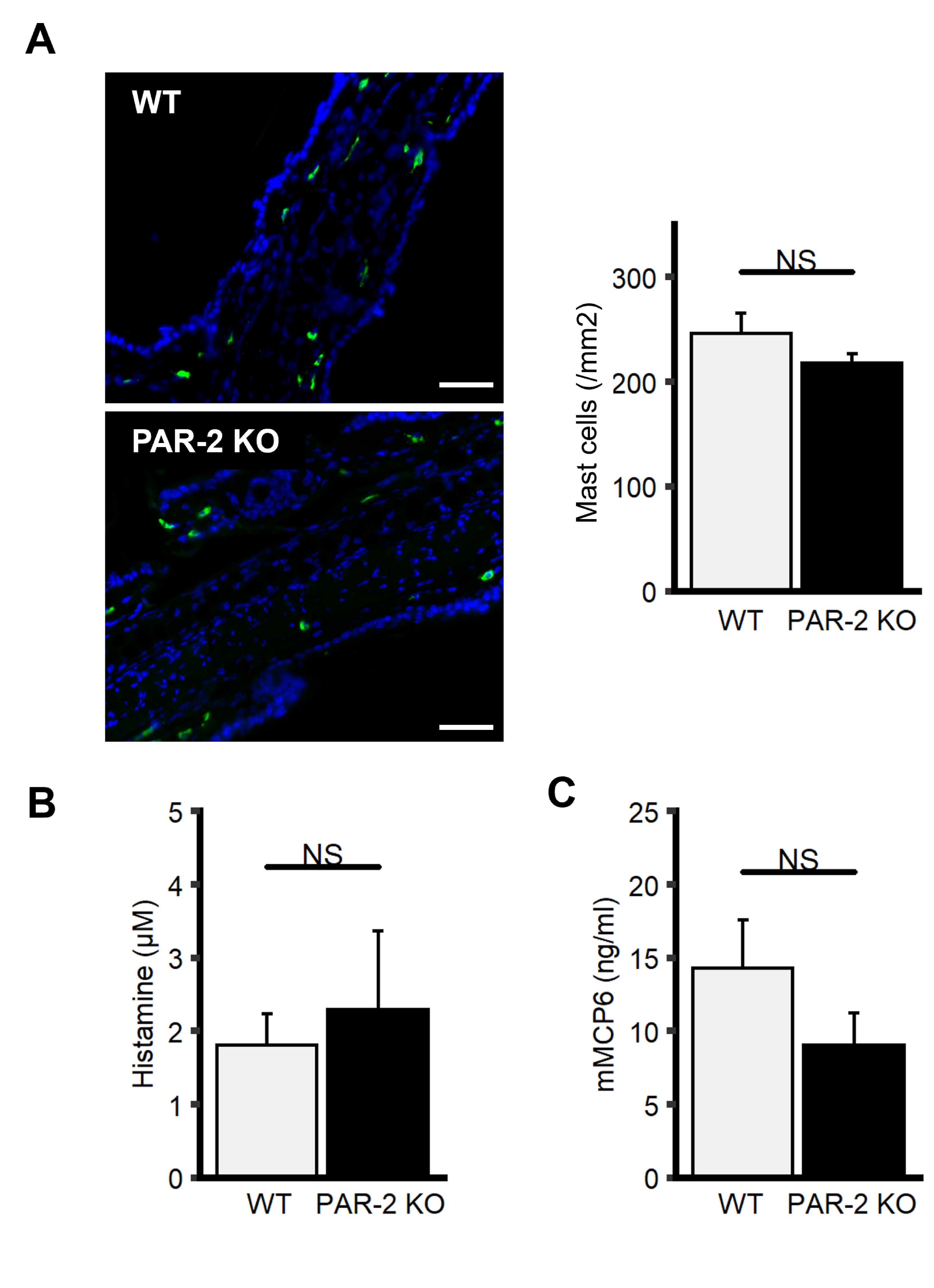

Supplement: S1 Fig — (A) Mast cells stained with FITC-avidin in the ears of mice (left) and the number of mast cells (right, n = 3). Magnification, ×200. Bar, 100 μm. (B) Serum histamine concentrations after C48/80 injection (n = 4). (C) Serum mMCP-6 concentrations after C48/80 injection (n = 4). NS, not significant. Data are presented as the mean ± SEM. (TIF) [file pone.0283915.s001.tif]

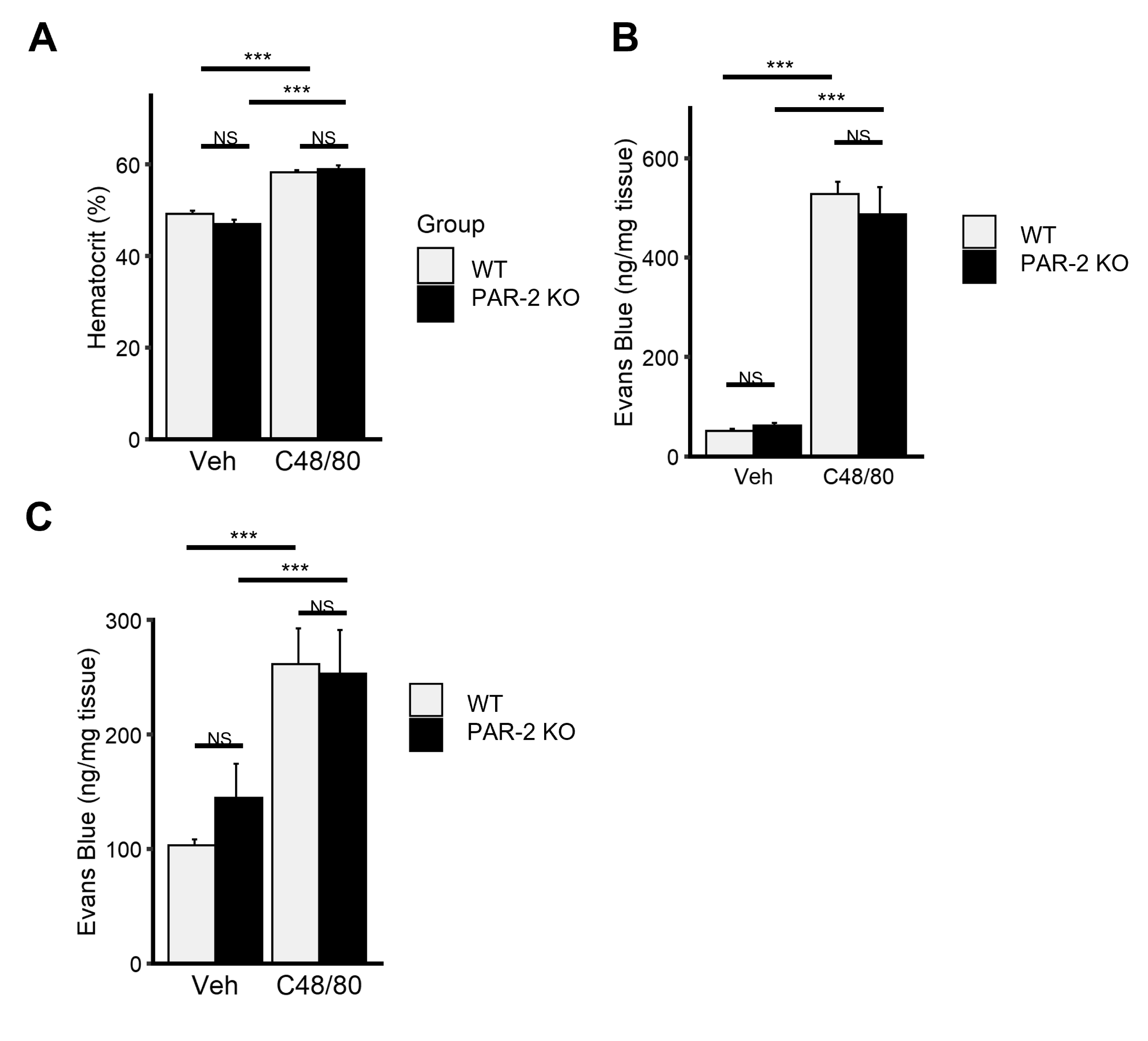

Supplement: S2 Fig — (A) Hematocrit after injection of C48/80 or vehicle in WT or PAR-2 KO mice (n = 4). (B) Evans blue dye leakage in the lungs after systemic injection of C48/80 or vehicle in WT or PAR-2 KO mice (n = 5). (C) Evans blue dye leakage in the ears after local injection of C48/80 or vehicle in WT or PAR-2 KO mice (n = 5). NS, not significant. ***P < 0.001. Data are presented as the mean ± SEM. (TIF) [file pone.0283915.s002.tif]
