# Supplementary material for: Protective role of protease-activated receptor-2 in anaphylaxis model mice
Source: PLoS One. 2024 Apr 18;19(4):e0283915. doi: 10.1371/journal.pone.0283915 (PMC11025949; doi:10.1371/journal.pone.0283915)

Fig 3B

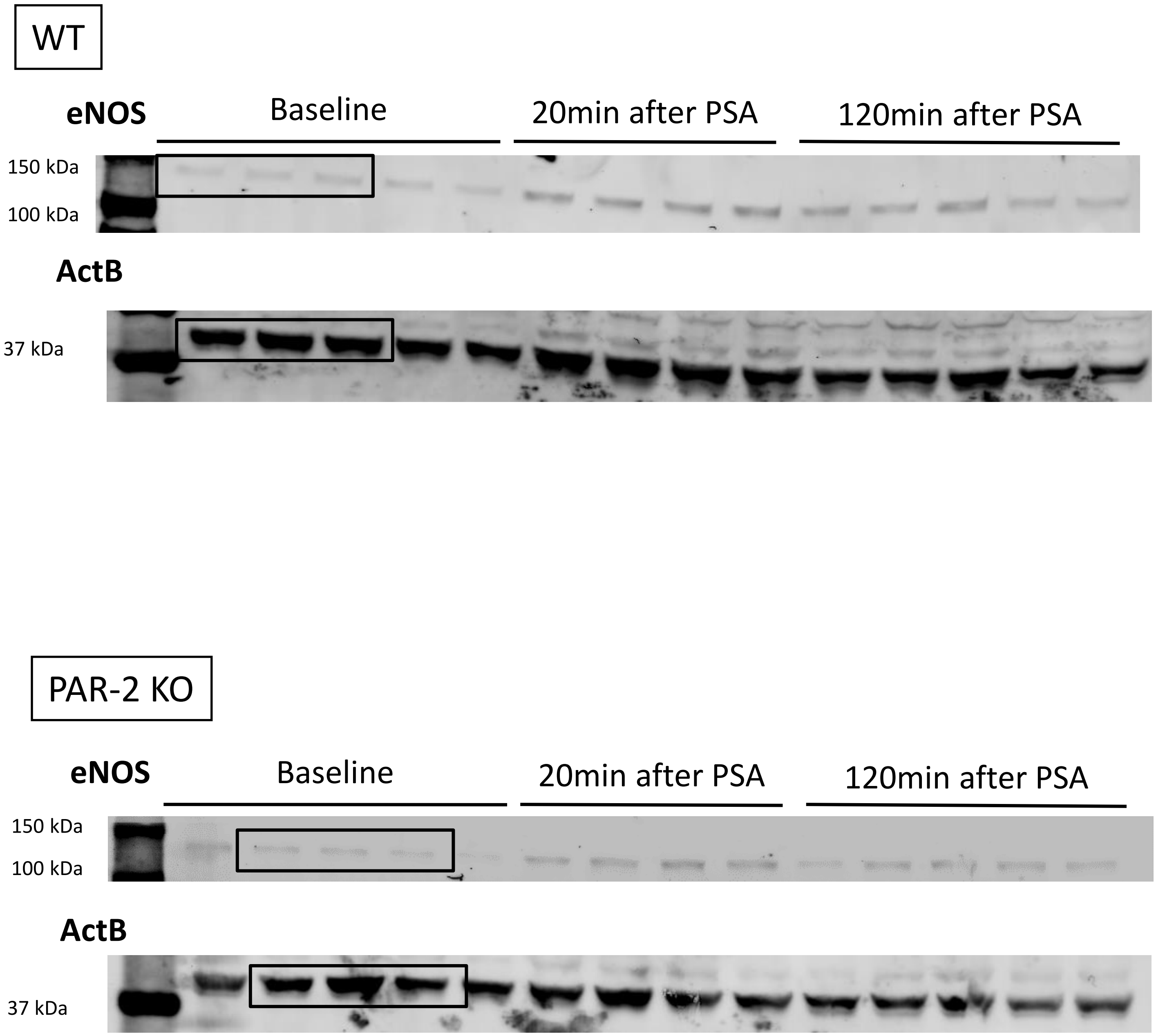
WT
eNOS
Baseline
20min after PSA
120min after PSA
150 kDa
100 kDa
ActB
37 kDa
PAR-2 KO
eNOS
Baseline
20min after PSA
120min after PSA
150 kDa
100 kDa
ActB
37 kDa

Fig 3B

【uncropped data】

p-eNOS

WT — PAR-2 KO

150 kDa
100 kDa

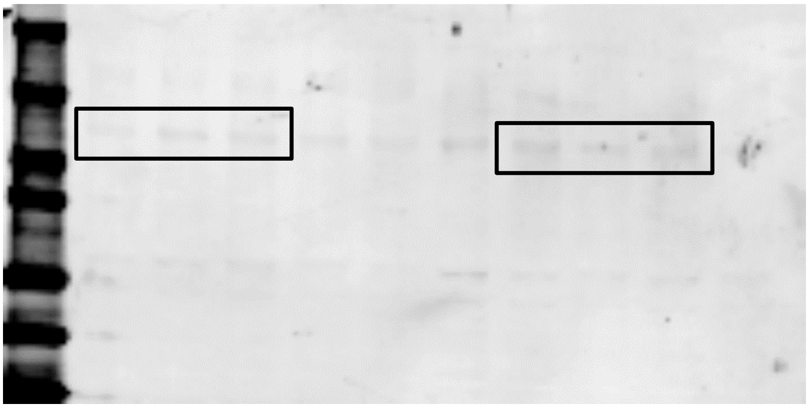

eNOS

150 kDa
100 kDa

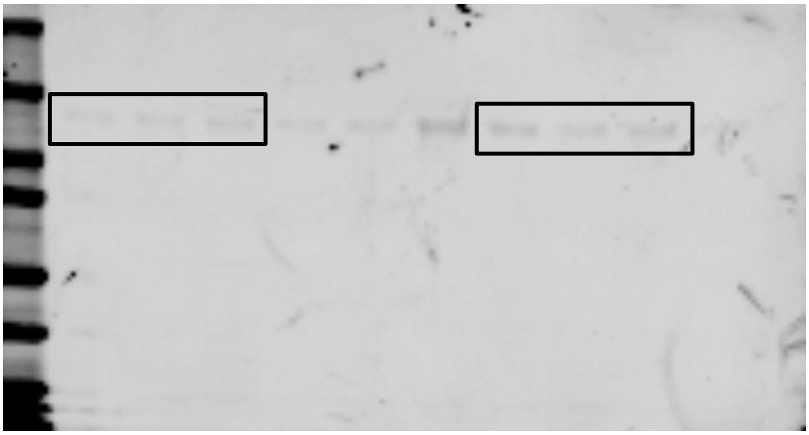

【cropped and contrast matched data】

p-eNOS

WT — PAR-2 KO

150 kDa
100 kDa

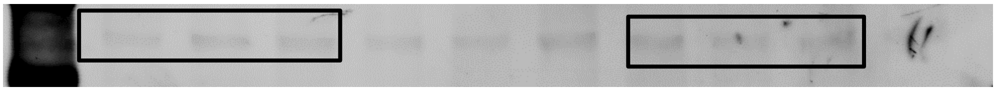

eNOS

150 kDa
100 kDa

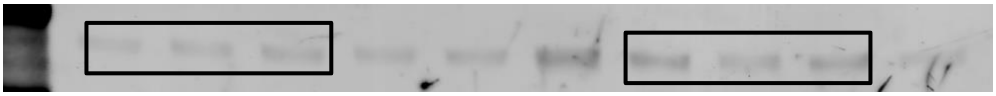

Fig 3C

【uncropped data】

eNOS

ActB

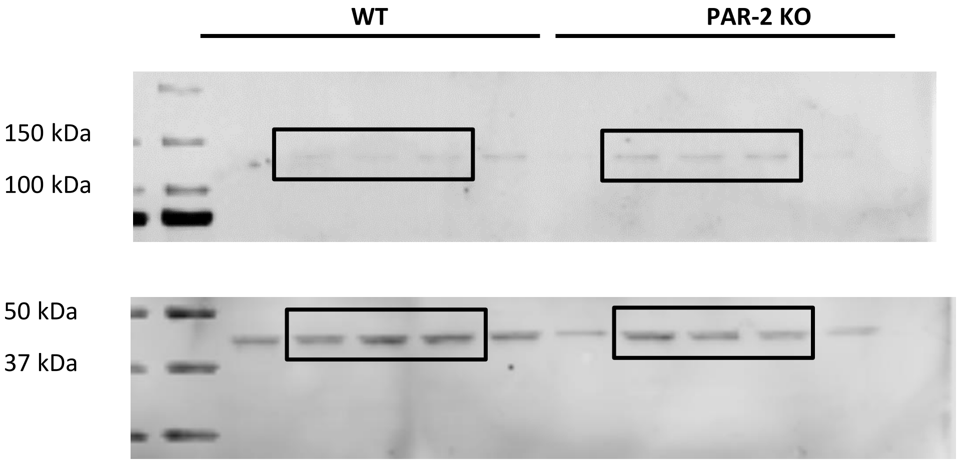

【cropped and contrast matched data】

eNOS

ActB

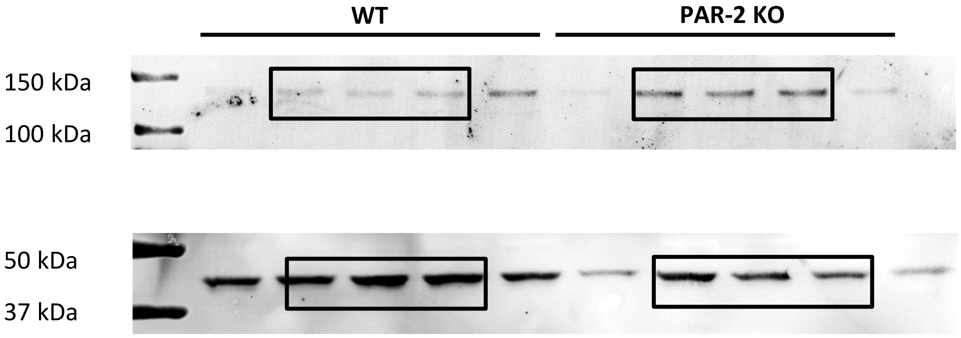

Fig 3C

【uncropped data】

WT PAR-2 KO

p-eNOS

150 kDa
100 kDa

eNOS

150 kDa
100 kDa

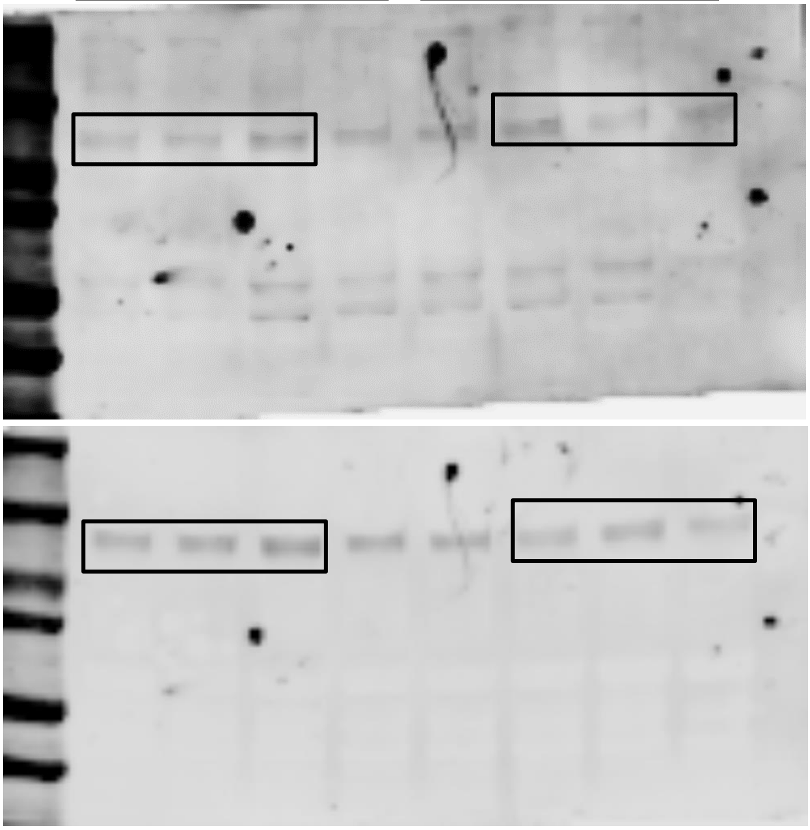

【cropped and contrast matched data】

WT PAR-2 KO

p-eNOS

150 kDa
100 kDa

eNOS

150 kDa
100 kDa

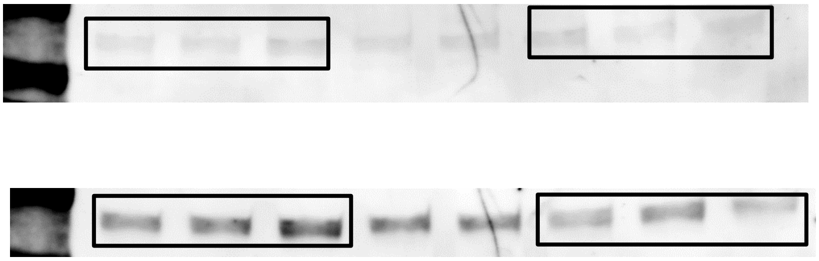

Fig 5B

【uncropped data】

p-eNOS

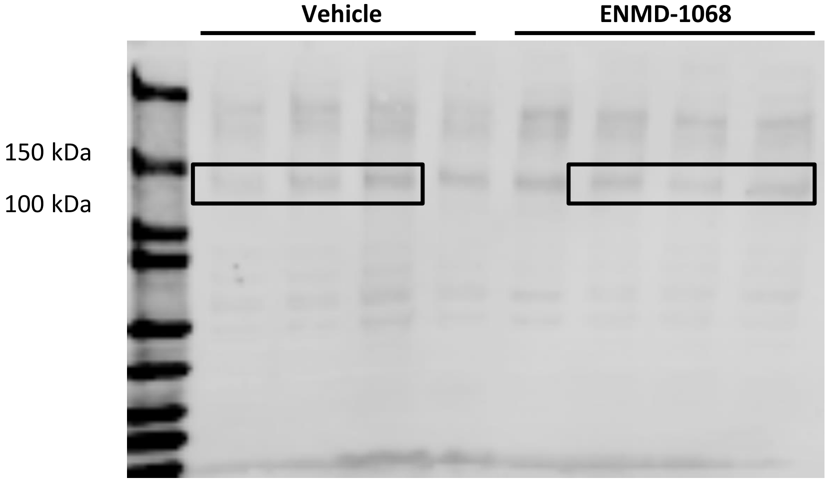

eNOS

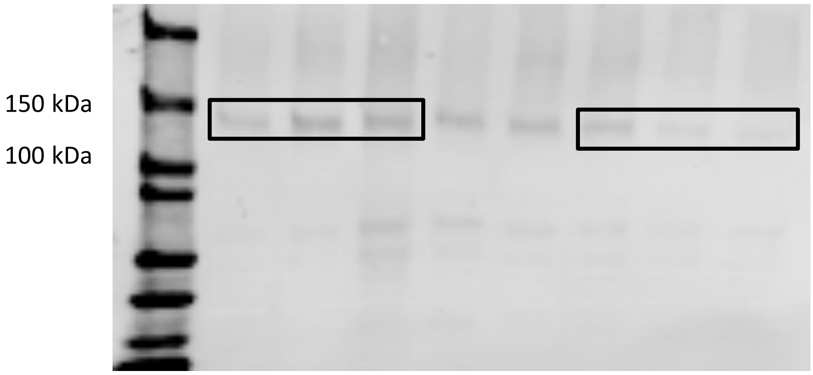

【cropped and contrast matched data】

p-eNOS

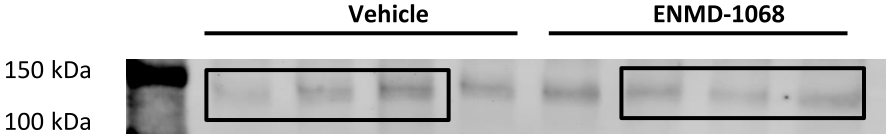

eNOS

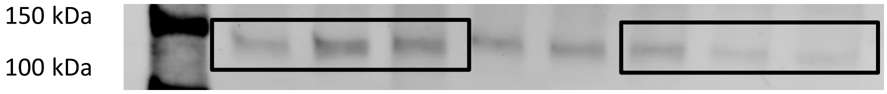

Supplement: S1 Data — (PDF) [file pone.0283915.s003.pdf]
