# Supplementary material for: Protective role of protease-activated receptor-2 in anaphylaxis model mice
Source: PLoS One. 2024 Apr 18;19(4):e0283915. doi: 10.1371/journal.pone.0283915 (PMC11025949; doi:10.1371/journal.pone.0283915)
Supplement: Attachment — Submitted filename: 240224_Response to Reviewer.docx [file pone.0283915.s004.docx]

Response to Reviewers

**・1. In Figure 3: The authors show that eNOS mRNA expression level was increased at 60 min (Fig. 3A) and eNOS protein expression level was increased at 120 min (Fig. 3C) in the lung of PAR-2-deficient mice after C48/80 administration, respectively. However, the peak temperature drop due to anaphylaxis was 15 min after C48/80 administration (Fig. 1B). If up-regulation of eNOS expression is responsible for the exacerbation of anaphylactic reactions in PAR-2-deficient mice, it likely occurred already before anaphylaxis was induced. Therefore, the authors have to include data or explanations in the manuscript that better illustrate this point.**

Thank you for pointing out the important point. We think that rapid eNOS phosphorylation occurs in early phase of anaphylaxis and decreases body temperature in anaphylaxis. Subsequently, a compensatory increase in eNOS protein expression may occur. We have included this discussion in Manuscript L338 onwards.

**・2. In Figure 4: Increased eNOS phosphorylation levels are a reasonable explanation for exacerbated anaphylactic reactions in PAR-2-deficient mice. Is enhanced eNOS activation also observed when the PAR-2 antagonist ENMD-1068 is administered to wild-type mice? In contrast, is it not observed when that is administered to mMCP-6-deficient mice?**

Thank you for your very pertinent questions. Additional experiments revealed that PAR-2 antagonism can also enhance eNOS activation in vivo during anaphylaxis (Fig 5B and Manuscript L291 onwards). On the other hand, as there was no difference in body temperature drop during anaphylaxis between mMCP-6 KO mice and WT mice, we believe there must be no difference in eNOS activation between both genotypes.

**3. In Figure 4: If possible, please also show the time-dependent changes in body temperature during IgE-dependent anaphylaxis in mMCP-6-deficient mice. I think many readers will be interested in that data.**

We are also interested in that point. There was no different in body temperature drop in IgE-independent anaphylaxis between WT and mMCP-6 KO mice (Fig 4D), suggesting that the role of mMCP-6 in anaphylaxis is not restricted to that of PAR-2. We are also interested in the role of mMCP-6 in anaphylaxis and further experiment, including IgE-dependent anaphylaxis, is necessary.

**Minor comments,**

**1. In Figure 4: It would be better to show serum mMCP-6 levels in wild-type mice and mMCP-6-deficient mice during anaphylaxis. In addition to the immunofluorescence images, please provide data that allow quantitative assessment of mMCP-6 gene deficiency**.

Thank you for your accurate comments. We added the result of plasma concentrations of mMCP-6 in WT and mMCP-6 deficient mice (Fig 4C). In WT mice, mMCP-6 was detected after C48/80 injection, on the other hand in mMCP-6 KO mice, mMCP-6 was not detected even in anaphylactic state.

Response to Editor’s comments

**1. We corrected the raw data of Figure 5B.**

As you pointed out, the uncropped data of Fig 5B was wrong one. We replaced this data with correct one. In addition, the annotation of cropped data was also incorrect. The annotation has been corrected. We are very sorry that we made such mistakes.

**2. We added the precise number of samples analyzed in Figure 3B and 3C**

In manuscript L260 & 265, we added the precise number of samples analyzed in Figure 3B and 3C.

**3.We updated the link to the raw data files in the cover letter.**

After updating the raw data, we updated the cover letter link to the raw data. The DOI was also written in cover letter.
